# Ultrasonography in Bell’s palsy: the elephant in the room

**DOI:** 10.1016/j.bjorl.2024.101433

**Published:** 2024-04-08

**Authors:** Ahmad J. Abdulsalam, Ameerah Alsaqobi, Murat Kara

**Affiliations:** a Hacettepe University Medical School, Department of Physical and Rehabilitation Medicine, Ankara, Turkey; b Mubarak Alkabeer Hospital, Department of Physical Medicine and Rehabilitation, Jabriya, Kuwait; c Physical Medicine and Rehabilitation Hospital, Department of Physical Medicine and Rehabilitation, Andalous, Kuwait

Dear Editor,

We read with interest the recently published article “Task force of the Brazilian Society of Otology — evaluation and management of peripheral facial palsy” by Pauna et al.^1^ We would like to congratulate the authors for their in-depth evidence-based review on the evaluation and management of peripheral facial palsy. However, we believe the utility of Ultrasound (US) in Bell’s palsy was overlooked.

The US imaging to assess peripheral nerve disorders is well established and has become one of the most useful modalities.^2^ Its diagnostic novelty and importance are sometimes being compared to electrodiagnostic studies.^3^ This is particularly true in peripheral nerve entrapment. Although electrodiagnostic tests are widely used, they are insufficient in detecting the exact location of nerve injury or the underlying cause. Moreover, morphological assessment of the nerve and surrounding structures is not possible.

From a diagnostic perspective, US may provide evidence of nerve edema spreading distally from the site of facial nerve damage, making it a useful tool for evaluating the main trunk of the facial nerve in Bell’s palsy (Fig. 1).^3^ The prognostic value of US in this condition has also been highlighted in the literature.^3^ In one study, US was found superior to electrodiagnostic tests in predicting good versus poor recovery at three months post onset of weakness.^4^ The direct evaluation offered by US is less time consuming and efficient, in contrast to other modalities that are commonly used during evaluation and follow-up of Bell’s palsy.

**Figure 1:**
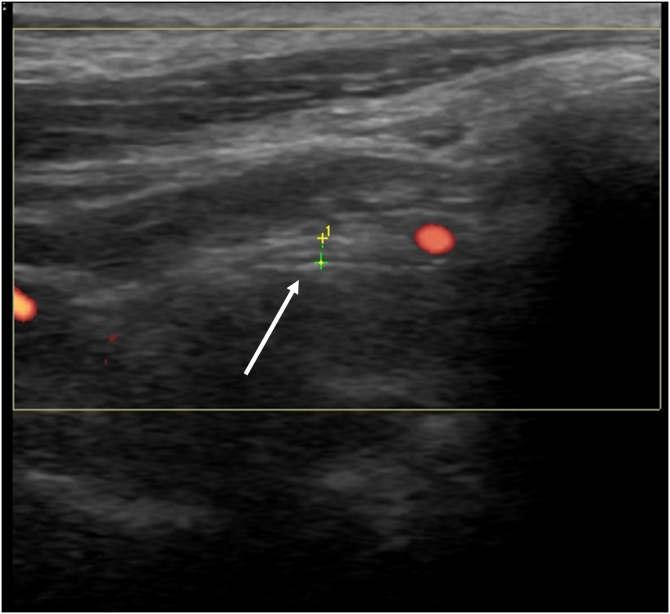
Ultrasound imaging/measurement (longitudinal view) of the facial nerve (white arrow) as they exit the stylomastoid foramen.

The utility of US in Bell’s palsy also extends to treatment. The validity as well as the reliability of US use in the management of facial nerve pathologies including Bell’s palsy has been documented in recent studies.^4^, ^5^ To conclude, we underscore the potential role of US examination in Bell’s palsy in two ways. First, US can confirm the presence of an inflamed facial nerve and rule out any local pathology. Secondly, the exact site for injection can be localized. This in turn helps reduce the required blood drug concentration of corticosteroids and contributes to better recovery and less side effects.^4^

## Conflicts of interest

The authors declare no conflicts of interest.

## Ethical statement

Not applicable.
